# Comparison of Ultrasonic Dissection and Suture Ligation for Mesoappendix in Laparoscopic Appendectomy

**DOI:** 10.7759/cureus.14316

**Published:** 2021-04-06

**Authors:** Muhammad Umar Qaiser, Aamna Nazir, Muhammad Sheharyar Khan, Hafiza Kiran Butt, Muhammad Anwar

**Affiliations:** 1 Surgery, District Headquarter (DHQ) Hospital, Rawalpindi, PAK; 2 Surgery, Holy Family Hospital, Rawalpindi, PAK; 3 Surgery, Rawalpindi Medical University, Rawalpindi, PAK

**Keywords:** laparoscopic appendectomy, suture ligation, mesoappendix, ultrasonic dissection

## Abstract

Objective

To compare the use of ultrasonic dissector and suture ligation for mesoappendix in laparoscopic appendectomy in terms of mean operative time and per-operative bleed.

Methodology

This was a randomized controlled trial conducted at Surgical Unit II, Holy Family Hospital over a period of six months. All patients with the diagnosis of acute appendicitis who presented in the emergency department of Holy Family Hospital on the call days of Surgical Unit II undergoing laparoscopic appendectomy were included in the study. After taking informed consent about the respective procedure, patients were allocated to two groups using computer-generated random numbers. Group A was operated using harmonic scalpel and in Group B suture ligation was done. Total time required to ligate the mesoappendix or to cauterize it using the harmonic scalpel was measured in minutes. Data was entered and analyzed in SPSS version 21.0 (IBM Corp., Armonk, NY).

Results

A total of 110 patients were enrolled in the study according to the inclusion criteria of the study. Patients were randomly divided into two equal groups. Patients in Group A were operated using harmonic scalpel whereas patients in Group B were operated by suture ligation for dealing the mesoappendix in laparoscopic appendectomy. Mean operative time to ligate the mesoappendix for Group A patients was 1.56 (SD = 0.68) minutes while that of Group B was 21.07 (SD = 2.84) minutes. There was no per-operative bleeding in case of Group A while its incidence was 24% of patients in Group B. Results were statistically significant.

Conclusion

The conclusion of the study is that the use of harmonic scalpel was better than suture ligation for ligating the mesoappendix in laparoscopic appendectomy in terms of mean operative time and per operative bleed, hence it's preferable over the later in laparoscopic appendectomy.

## Introduction

Appendicitis, a frequent cause of acute abdominal pain, is considered as one of the most common indications for emergency abdominal surgery [1]. Laparoscopic appendectomy was performed by Kurt Semm in 1983 and with this he set a new breakthrough in minimal access surgery. Although the procedure has yet not been recognized as the ‘Gold Standard’ for treatment of appendicitis, it has gained popularity due to decreased operative time, better cosmesis, faster recovery and shorter hospital stays [2, 3]. Conservative treatment for appendicitis has been compared with appendectomy, but appendectomy and more specifically, open appendectomy prevails as the procedure of choice for treating appendicitis. However, laparoscopic techniques are still being evaluated for their effectiveness [4].

There are many techniques which further modify laparoscopic appendectomy techniques. Basic steps of laparoscopic appendectomy include: port insertion, identification of appendix, separation of mesoappendix and separation of the base. However, techniques employed at handling the mesoappendix and separation of base greatly vary [5]. There are numerous methods available for dissecting the mesoappendix including using an endostapler, LigaSure (Covidien, Boulder, CO), ultrasonic dissector (Ethicon Endosurgery, Cincinnati, OH), or electro cautery. Cutting the appendix mesentery by laparoscopic scissors and suturing is time consuming. Extracorporeal suturing requires experience and technique in order to perform it. With a steep learning curve, many surgeons feel difficulty in using this technique [6].

Ultrasonic dissector, on the other hand, is considered as a more reliable method of ligation of mesoappendix because of precision and accuracy of the dissection, as well as reduced expertise. An ultrasonic dissector is an ultrasound activated disposable knife which transforms longitudinal movement by its piezoelectric element leading to cutting and sealing of edges at the same time. The instrument is similar to surgical diathermy but superior in terms of ability to cut through thicker tissue and production of lesser smoke. However, it is not easily maneuverable, costly and requires more time [7, 8].

Considering the marked difference between the different techniques of ligating the mesoappendix, there has been a need in literature to identify by comparative analysis which technique is the more superior. Since, minimal work has been done to compare operative time and per-operative bleeding between suture ligation and ultrasonic dissection, we decided to compare the two techniques based on these factors. Hence, the investigators of this study conducted a pilot study of 10 patients to compare ultrasonic dissector (n = 5) with suture ligation (n = 5), in which operative time in both the groups was 1.90+0.937 and 24.30+6.028, respectively. Considering the difference between both the groups, we conducted a study with a larger population to determine whether a difference between the two groups exists at a significant level or not.

## Materials and methods

This was a prospective randomized controlled trial that was carried out in the Department of Surgery, Unit II, Holy Family Hospital from January 2018 to December 2019. Prior to initiation of the trial, approval was sought from the Institutional Research Forum of Rawalpindi Medical University. After approval, the patients presenting in the OPD were scrutinized and inducted as a part of the study. Before the recruitment into the study, all the patients were informed about the risks involved in the study, after which informed consent was obtained. Patients were scrutinized on the basis of the following criteria:

Inclusion criteria

1. 15 - 45 years

2. Any gender

3. Patient willing to undergo laparoscopic procedure

4. Acute appendicitis confirmed on histopathological analysis

5. Alvarado Score ≥5 with positive ultrasound findings

Exclusion criteria

1. On the basis of history and clinical evaluation

2. Any other intra-abdominal pathology other than acute appendicitis, e.g., appendicular mass, appendicular abscess, malrotated gut, tuberculosis, carcinoid tumor, pelvic inflammatory disease, ovarian tumors, ceacal mass, etc.

3. Conversion to open technique

4. Contraindication to laparoscopy

5. Patients declared unfit for surgery during preoperative assessment

All patients with the diagnosis of acute appendicitis presented in the emergency department of Holy Family Hospital on the call days of Surgical Unit II undergoing laparoscopic appendectomy were included in the study.

Diagnostic criteria

The diagnosis of acute appendicitis was based on the Alvarado score shown in Table 1.

**Table 1:** Alvarado Scoring System

	Score
Symptoms	
-Migratory RIF Pain	1
-Anorexia	1
-Nausea and Vomiting	1
Signs	
-Tenderness	2
-Rebound Tenderness	1
-Elevated Temperature	1
Laboratory Findings	
-Leucocytosis	2
-Shift to the Left (segmented neutrophils)	1

Patients having a score of 7 or greater were considered as confirmed diagnosis of acute appendicitis. While those patients who had a score of 5 or 6, were considered suspicious of acute appendicitis. All patients with a score 5 or greater underwent urgent ultrasound to either confirm the diagnosis or rule out complications. Ultrasound findings suggestive of acute appendicitis were:

1. Aperistaltic, non-compressible, dilated appendix (>6 mm out diameter)

2. Appendicolith

3. Distinct appendiceal wall layers

4. Echogenic prominent pericaecal fat

5. Periappendiceal fluid collection

6. Target appearance (axial section)

Randomization technique

Patients were allocated to two groups (Group A: Ultrasonic dissector, Group B: Suture ligation) using computer generated random numbers. Allocation concealment was carried out with the sequentially numbered, opaque, sealed envelopes (SNOSE) technique. The envelope was opened after the patients were induced under general anesthesia hence, ensuring blinding in the study. Principal investigator collected the data during the surgical procedure using a self-designed performa. The procedure technique was denoted numerical figures of 1 and 2, and data entry and analysis was performed by a co-investigator who was kept blinded of the type of ligation technique.

Surgical procedure

Group A was operated using ultrasonic dissector to ligate the mesoappendix and in Group B suture ligation was done. All patients were given pre-operative analgesics and antibiotic, in the form of intramuscular diclofenac sodium 30 mg, injection ceftriaxone 1 gram and injection metronidazole 500 milligrams, respectively. Both the groups underwent through the same technique of laparoscopic technique.

A day prior to surgery, preoperative assessment was done of each patient for surgical procedure fitness. In Operation Theater, patients were positioned supine in Trendelenburg position and under aseptic measures Foley’s catheter was introduced for voiding of bladder to provide better visualization by bladder decompression. Trocar (10 mm) was placed in the umbilicus. Additional 5 mm two ports were placed in the suprapubic place (2 cm above pubic symphysis) and left lower quadrant (3 cm superior and medial to the anterior superior iliac spine). Ultrasonic dissector (Group A) or extracorporeally prepared knots (Group B) were introduced through the port in left lower quadrant. The appendix stump was closed in both the groups by the endoloop method. The appendix was transected and retrieved (grasper) by similar technique in both the groups. Surgery was performed by Assistant Professor, Associate Professors or Professors of Surgery in Surgical Unit II, Holy Family Hospital.

Post-operative sample of appendix was sent for histopathological analysis and pathologies other than acute appendicitis were excluded from the data base before final data analysis.

Outcome variables

Operative Time

Operative time was defined as the time taken to separate the mesoappendix. Time was initiated as soon as the ultrasonic dissector or suture was introduced through the port and was noted down till the mesoappendix was separated by either of the two techniques being studied. This variable was measured in minutes.

Blood Loss

Blood loss was qualitatively defined as either occurring or not (yes/no) according to the principal investigator’s visual assessment. Blood loss occurring during the time when dealing with mesoappendix was only considered.

Data Analysis Technique

Data was entered and analyzed in SPSS version 21.0 (IBM Corp., Armonk, NY). Mean and standard deviation was calculated for quantitative variables like age, operative time (minutes). Frequency and percentages were calculated for qualitative variables like gender of patient, per-operative bleeding. Independent sample t-test was used to compare operative time (minutes) in both the groups. Chi-square test was used to compare per-operative bleeding in both the groups. P < 0.05 was taken as level of significance.

## Results

**Figure 1:**
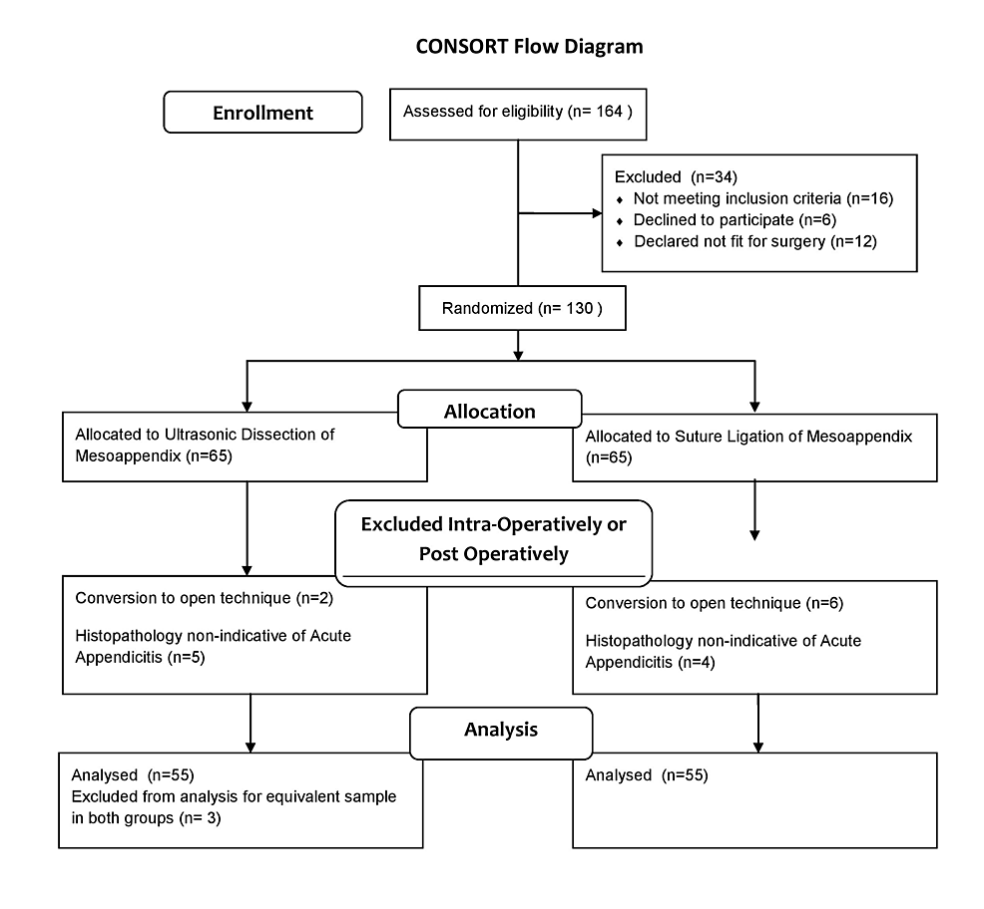
Consort flow diagram

Total 163 patients were enrolled in the study according to the inclusion and exclusion criteria of the study as shown in Figure 1. Patients were randomly divided into two equal groups. Patients in Group A were operated using ultrasonic dissector whereas patients in Group B were operated by suture ligation for dealing the mesoappendix in laparoscopic appendectomy. A total of 110 patients were included in the final data analysis as shown in Figure 1.

Descriptive statistics of age (years) of patients were calculated in terms of mean and standard deviation. Mean age in both the groups were 23.75+6.55 and 24.27+7.00 respectively, as shown in Table 2. Distribution of gender was also calculated in our study in terms of frequency and percentages of male and female patients. In our study, majority of the patients were male, i.e. 33 (60.0%) and 29 (52.7%) male patients in both the groups, whereas 22 (40.0%) female patients were in Group A which was operated by ultrasonic dissector and 26 (47.3%) female patients were operated by suture ligation in Group B, as shown in Table 2.

**Table 2:** Distribution of age and gender

		Group A - Ultrasonic Dissector	Group B - Suture Ligation of Mesoappendix	Pearson Chi Square/Independent T Test
Gender (Frequency, percentage)	Male	33 (60.0%)	29 (52.7%)	0.841
	Female	22 (40.0%)	26 (47.3%)	
Age (Mean, Standard Deviation, Range)		23.75±6.55 (21-63)	24.75±7.01 (19-56)	0.642

Per-operative bleeding was also calculated in terms of frequency and percentages of preoperative bleeding. In Group B it was 24 (43.6%) whereas, no patient was found to have preoperative bleeding in Group A. Chi-square test was used to compare per-operative bleeding in both the groups which was statistically significant (p-value <0.001) in both the groups, which showed use of ultrasonic dissector was better than suture ligation for dealing the mesoappendix in laparoscopic appendectomy in terms of per-operative bleeding, as shown in Table 3.

**Table 3:** Comparison of per-operative bleeding between both the groups

		Group A - Ultrasonic Dissector	Group B - Suture Ligation of Mesoappendix	Pearson Chi Square
Per-operative Bleeding	Yes	0 (0.0%)	24 (43.6%)	p-value < 0.001
	No	55 (100.0%)	31 (56.4%)	
Total		55 (100.0%)	55 (100.0%)	

Comparison of operative time was recorded in terms of minutes. Mean operative time (min) in both the groups was 1.56+0.68 and 21.07+2.84, respectively. Independent samples t-test was used to compare operative time (min) in both the groups which was statistically significant (p-value <0.001) in both the groups, which showed use of ultrasonic dissector was better than suture ligation for dealing the mesoappendix in laparoscopic appendectomy in terms of operative time, as shown in Table 4.

**Table 4:** Comparison of operative time between both the groups

	Group A - Ultrasonic Dissector	Group B - Suture Ligation of Mesoappendix	Independent T Test
Mean Operative Time (Minutes)	1.56 ± 0.688	21.07 ± 2.847	p-value < 0.001

## Discussion

Emerging trends are getting inclined towards laparoscopic approach for appendectomy because of reduced operative time and better cosmetics. It is now being considered as a safe and easier approach. A study by Ward et al. showed laparoscopic approach to have better clinical outcomes. In comparison to international series, mortality rates and complications remained minimal in patients going under laparoscopic appendectomy but with added advantages as mentioned before [9]. Hence, exploring the option of laparoscopic appendectomy has become a need of time.

Ultrasonic dissector has been used in multiple surgeries and has been superior to conventional techniques. It proved to reduce operative time and blood loss in total thyroidectomy when compared against conventional suturing in a study done by El Sherpiny [10]. Similarly ultrasonic dissector was compared with electrocautery in hemorrhoidectomy, and was proven to be associated with decreased post-operative pain, decreased intra-operative bleeding, decreased bleeding time and lastly decreased lateral thermal damage [11]. Hence, the usage of ultrasonic dissector has had advantages in different surgeries.

A similar study to ours but assessing safety was conducted by Yavuz et al. and it proved that ligation and stump closure with ultrasonic dissector was as safe as it was with silk sutures [12]. However, this study did not produce any measurable outcomes unlike our study. Our study was also comparable to a study done by Gupta et al. in which a comparison was highlighted in securing hemostasis and sealing the base between suture ligation and ultrasonic dissector, in which the total operative time was 43.43 ± 6.7 minutes and 28.46 ± 7.19 minutes respectively, a total difference of 14.97 minutes [13]. While we only measured the total time to ligate the mesoappendix, showing a difference of 19.51 minutes; group A and group B having 1.56 ± 0.688 and 21.07 ± 2.847 minutes, respectively. Adding further, our study also reports the incidence of per-operative bleeding which was not reported in the aforementioned study. The methodology of our study was different from this study as we secured the base with endoloop in both the groups. The usage of ultrasonic dissector due to its low complication rates and decreased operative time has been recommended by another study by Martin Del Olmo et al. [14].

Other techniques have also been employed in the treatment of mesoappendix. A popular technique used worldwide is of using endoclips. A study done in 2018 stated that endoclip is a relatively costly technique [15]. The available endoclips in our setup are usually titanium based which makes them expensive to be used. On the other hand, ultrasonic dissector is considered a one-time investment which can be used multiple times. Hence, endoclip is not a financially viable option to be used in developing countries. As a result, one-time investment techniques such as LigaSure, monopolar cautery and ultrasonic dissector are relatively preferred. Adding further, the availability of new instruments is relatively limited to developed cities in developing countries, hence if laparoscopic appendectomies are conducted, a vast majority uses extra-corporeal or intra-corporeal suturing techniques. As a result, studies like this should be conducted which document the marked difference of using new instruments compared to suturing techniques.

Among the new techniques available, ultrasonic dissector is preferred in some studies. In study published in 2020, combinations of different techniques were analysed in a total of 846 patients which yielded results in the favor of usage of ultrasonic dissector and LigaSure as techniques to secure hemostasis during laparoscopic appendectomy [16]. Although they cost relatively more but considering the reduced operative complications, these techniques should be made available for better operative outcomes. Similarly, a comparison between endoloop and LigaSure was made; each having operative time of 54 minutes and 41 minutes, respectively [17]. This yet again, proved that newer techniques such as LigaSure are time-saving alternatives. However, when compared with LigaSure and thermal fusion technology, ultrasonic dissector produced less lateral thermal damage during laparoscopic appendectomy according to a study done in children from 2013 to 2015 [18]. Our study did not investigate the usage of LigaSure or monopolar cautery, but compared suture ligation and ultrasonic dissection. However, this study does clarify that it is viable to move and progress to newer techniques within Pakistan which can improve patient outcomes and reduce pressure on healthcare resources by reducing operative time.

## Conclusions

After an analysis of 110 patients undergoing suture ligation and ultrasonic dissector stasis of the mesoappendix, we conclude that usage of ultrasonic dissector was superior compared to suture ligation during laparoscopic appendectomy. This was significantly proven by reduced per-operative bleeding using Chi Square Test, and reduced per-operative time using Student's t-test.

We would like to recommend further studies to be undertaken with more variables such as post operative complication rates, pain scores, incidence of post operative infections, conversion rates and recovery time to get a better conclusion as to which technique is ultimately most beneficial for the patients. Moreover, further analysis should be done to discuss the cost effectiveness of this technique (Ultrasonic Dissector). Adding further, comparative studies should be held to compare ultrasonic dissector with LigaSure and monopolar cautery in laparoscopic appendectomy.
